# Supplementary material for: Deletion of wfs1 Impairs Oligodendrocyte Precursor Cells Dorsal Distribution and Myelination Through the wfs1-hmgcs1 Axis in Zebrafish
Source: Biology (Basel). 2026 Aug 16;15(16):1402. doi: 10.3390/biology15161402 (PMC13510151; doi:10.3390/biology15161402)
Supplement: Supplementary file 1 [file biology-15-01402-s001.zip › File S1-Transcriptome sequencing data and differentially expressed genes (DEGs) in wfs1-- zebrafish at 5 dpf/2.KEGG/wfs1KOvsWT/ALL/src/dre00590.html]

dre00590


- dre:393592

- Up regulated genes

393592(3.73058515777409)
- dre:797309

- Down regulated genes

797309(-2.56336508517195)
- dre:399485

- Down regulated genes

399485(-1.02621748606863)

- dre:393592

- Up regulated genes

393592(3.73058515777409)
- dre:797309

- Down regulated genes

797309(-2.56336508517195)
- dre:399485

- Down regulated genes

399485(-1.02621748606863)

- dre:393592

- Up regulated genes

393592(3.73058515777409)
- dre:797309

- Down regulated genes

797309(-2.56336508517195)
- dre:399485

- Down regulated genes

399485(-1.02621748606863)

- dre:393592

- Up regulated genes

393592(3.73058515777409)
- dre:797309

- Down regulated genes

797309(-2.56336508517195)
- dre:399485

- Down regulated genes

399485(-1.02621748606863)

- dre:393592

- Up regulated genes

393592(3.73058515777409)
- dre:797309

- Down regulated genes

797309(-2.56336508517195)
- dre:399485

- Down regulated genes

399485(-1.02621748606863)

- dre:393592

- Up regulated genes

393592(3.73058515777409)
- dre:797309

- Down regulated genes

797309(-2.56336508517195)
- dre:399485

- Down regulated genes

399485(-1.02621748606863)

- dre:393592

- Up regulated genes

393592(3.73058515777409)
- dre:797309

- Down regulated genes

797309(-2.56336508517195)
- dre:399485

- Down regulated genes

399485(-1.02621748606863)

- dre:393592

- Up regulated genes

393592(3.73058515777409)
- dre:797309

- Down regulated genes

797309(-2.56336508517195)
- dre:399485

- Down regulated genes

399485(-1.02621748606863)

- dre:559020

- Up regulated genes

559020(1.27287729194892)

- dre:406370

- Up regulated genes

406370(7.33922178722892)

- Down regulated genes

406370\_1(-1.88308518916857)
- dre:560649

- Up regulated genes

560649(1.22362655394035)
- dre:767688

- Down regulated genes

767688(-1.20360432923595)
- dre:100148818

- Down regulated genes

100148818(-1.62621488580639)

- dre:406575

- Up regulated genes

406575\_1(1.68114903804516)

- dre:559020

- Up regulated genes

559020(1.27287729194892)

- dre:406449

- Up regulated genes

406449\_1(6.49480297810026)

- Down regulated genes

406449(-7.82321330212385)

- dre:767759

- Down regulated genes

767759(-1.52170076114492)

- dre:393297

- Up regulated genes

393297(1.19337638849417)

- dre:393592

- Up regulated genes

393592(3.73058515777409)
- dre:797309

- Down regulated genes

797309(-2.56336508517195)
- dre:399485

- Down regulated genes

399485(-1.02621748606863)

- dre:393297

- Up regulated genes

393297(1.19337638849417)

- dre:494108

- Up regulated genes

494108(2.34015565356837)

- Down regulated genes

494108\_1(-6.03454981408502)
- dre:566996

- Up regulated genes

566996(3.78724584427087)

- Down regulated genes

566996\_1(-5.26334001513029)

- dre:494108

- Up regulated genes

494108(2.34015565356837)

- Down regulated genes

494108\_1(-6.03454981408502)
- dre:566996

- Up regulated genes

566996(3.78724584427087)

- Down regulated genes

566996\_1(-5.26334001513029)

Close
